# Late Pregnancy is a Critical Period for Changes in Phosphorylated Mitogen‐Activated Protein Kinase/Extracellular Signal‐Regulated Kinase 1/2 in Oxytocin Neurones

**DOI:** 10.1111/jne.12398

**Published:** 2016-08-19

**Authors:** G. K. Chandaka, L. Wang, S. Senogles, W. E. Armstrong

**Affiliations:** ^1^ Department of Anatomy and NeurobiologyUniversity of Tennessee Health Science CenterMemphisTNUSA; ^2^ Microbiology, Immunology and BiochemistryUniversity of Tennessee Health Science CenterMemphisTNUSA; ^3^ Neuroscience InstituteUniversity of Tennessee Health Science CenterMemphisTNUSA

**Keywords:** oxytocin, vasopressin, pERK1/2, supraoptic nucleus, paraventricular nucleus

## Abstract

The physiological demands of parturition and lactation lead to the increased pulsatile release of oxytocin (OT) into the circulation from the neurohypophysial axons of OT neurones in the supraoptic (SON) and paraventricular (PVN) nuclei. These states of increased OT release are accompanied by a significant plasticity in magnocellular OT neurones and their synaptic connections, and many of these changes require activation of a central OT receptor. The mitogen‐activated protein kinase/extracellular signal‐regulated kinase pathway (MAPK/ERK) is assumed to be up‐regulated in the PVN during lactation, and many of the effects of OT in peripheral and brain tissue are mediated through a MAPK/ERK pathway. The present study investigated whether this pathway is altered in the SON and PVN during late pregnancy [embryonic day (E)20–21], which is a critical period for OT plasticity induction, and for lactation, when plastic changes are sustained. Based on immunoreactivity for phosphorylated ERK1/2 (pERK1/2), the results suggest an enhanced activation of MAPK/ERK pathway in OT neurones specifically during late pregnancy in both the SON and PVN. Although immunoblots from the SON confirm this pregnancy‐associated up‐regulation in late pregnancy, they also suggest enhancement into lactation as well. Together, the results suggest an important role for the MAPK/ERK pathway during reproductive changes in the SON and PVN.

One of the key neurohypophysial hormones, oxytocin (OT), is synthesised and released from the magnocellular cells (MNCs) of the supraoptic nucleus (SON) and paraventricular nucleus (PVN). Although best known to activate peripheral OT receptors (OTRs) mediating the induction of labour through uterine contractions, and the mammary myoepithelial cell contractions necessary for milk ejection during lactation 1, OT also acts as a peptidergic neurotransmitter in the brain 2, which contains an abundance of OTRs 3. In the SON and PVN, OT is released from the dendrites and somata 4, 5 to act on OTRs on OT neurones themselves. This autoregulatory activity of OT is assumed to be critical to the induction of the normal OT bursting patterns observed during milk ejection 6, 7.

The activity of OT in the brain is likely conditioned by changes in OTRs that occur in late pregnancy after the fall of progesterone 8, a time when local OT secretion itself becomes significantly elevated near parturition 9. Studies also showed that central OTR stimulation during mid and late gestation is essential for the normal OT secretory response during lactation, thus assuring the healthy development of the offspring 10, 11. Central OTR activation during pregnancy is also necessary for plasticity observed in intrinsic 12 and synaptic properties 13 of OT neurones. The fall of progesterone during the last few days of pregnancy may be critical for many OT‐related changes in the centrasl nervous system 8, 14, as well as in the induction of maternal behaviours 15.

There is increasing evidence that some of the effects of OT in the brain may be mediated through an mitogen‐activated protein kinase (MAPK)/extracellular signal‐regulated kinase (ERK) pathway that also has been implicated in some peripheral actions of OT 16. For example, centrally released OT improves spatial memory in the hippocampus of lactating rats through the phosphorylation of ERK1/2 17. There are also complex alterations in ERK1/2 signalling in the PVN during lactation, suggesting that this pathway might be involved in the regulation of anxiety‐like behaviour in female rats 18. Furthermore, both OT and vasopressin (VP) neurones exhibit an increased phosphorylated ERK1/2 (pERK1/2) in response to osmotic challenge 19 and pERK1/2 is selectively increased by psychological and systemic stressors in a subset VP neurones that contain interleukin‐6 20. In the present study, we investigated whether pERK1/2 varied during the reproductive cycle in the PVN and SON using immunohistochemistry and western blotting. The results suggest late pregnancy [embryonic day (E)20–21] is a critical period for pERK1/2 elevation in the SON and PVN, specifically within OT neurones.

## Materials and methods

### Animals

Adult Sprague–Dawley albino female rats (virgins), pregnant rats (E18–19), late‐pregnant rats (E20–21) and lactator rats (day 8 lactation) were used (Harlan Labs, Indianapolis, IN, USA) for both western blotting and immunohistochemical analyses. The rats had free access to food and water in the cages housed in a room under a 12 : 12 h light/dark cycle. The Institutional Animal Care and Use Committee approved all protocols used in the study.

### Immunohistochemistry

The rats were deeply anaesthetised with sodium pentobarbital (50 mg/kg) and perfused through the heart with cold 4% paraformaldehyde and 0.2% picric acid in 0.01 m phosphate‐buffered saline (PBS). The brains were excised and fixed overnight. Hypothalamic slices (50 μm) were prepared with a vibrating microtome (VT1000 Leica, Bannockburn, IL, USA). The slices were rinsed off fixative with several changes of PBS containing 0.5% Triton X‐100 (PBST), then incubated for 24–48 h at 4 °C for double‐labelling with rabbit anti‐pERK1/2 (dilution 1 : 1000, Catalogue number 4370S, Cell Signaling, Danvers, MA, USA) + anti‐OT antibody (PS36 or PS38) raised in mouse against OT‐NP (Oxytocin‐Neurophysin, dilution 1 : 500, a gift from Dr Harold Gainer, NIH, Bethesda, MD, USA). This experiment was repeated for VP neurones, with sections incubated in mouse anti‐pERK1/2 (dilution 1 : 1000, Catalogue number 5726S, Cell Signaling) + anti‐VP antibody (dilution 1 : 20 000) raised in rabbit against VP‐neurophysin (a gift from Dr Alan Robinson, UCLA, Los Angeles, CA, USA). The sections were rinsed in PBST and incubated in a cocktail of secondary antibodies for 3 h at room temperature. The secondary antibodies used were goat‐anti‐mouse (Alexa Flour 488 nm; Invitrogen, Carlsbad, CA, USA) and goat‐anti‐rabbit (Alexa Fluor 568 nm, Invitrogen) conjugated immunoglobulin G at a dilution of 1 : 200 with PBST. Rinsed sections were mounted on a glass slide and cover‐slipped using a polyvinyl alcohol (PVA) solution containing 6 g/25 ml glycerol, 2.4 g/25 ml PVA, 0.625 g/25 ml of the anti‐fade reagent 1,4 diazabicyclo[2.2.2]octane, brought to 25 ml with 6 ml dH_2_O and 12 ml PBS (all reagents, Sigma‐Aldrich, St Louis, MO, USA).

Tiled images were acquired from the SON or PVN with a Zeiss 710 confocal microscope (Carl Zeiss, Oberkochen, Germany) using a  × 20 objective (0.8 n.a.), with optical section oversampling of approximately 1 μm. The percentages of magnocellular OT and VP neurones that co‐localised with pERK1/2 in the PVN and SON were estimated from counts made in these confocal stacks with Zen (Carl Zeiss) or imagej (NIH, Bethesda, MD, USA). Antibody penetration was approximately 12–15 μm, and each section was tested for penetration depth of each antibody before sampling. Each neurone counted was observed in multiple optical sections to avoid double counts and, similarly, counts were made from slices separated by at least 50 μm to avoid counting the same neurone in two sections. When sampling ranged from the rostral to caudal borders of each nucleus in all groups, we did not estimate the total number of neurones stained for any of the antibodies, focusing only on VP or OT neurones for which the soma was within the focal plane and determining whether they co‐localised pERK1/2. Slides were coded by one investigator and counted blind by a second. The number of neurones sampled was:

OT neurones, PVN: virgin (3156), E18–19 (3161), E20–21 (3585), lactators (3377) and SON: virgin (2653), E18–19 (3640), E20–21 (3368), lactators (2866) (n = 6 animals).

VP neurones, PVN: virgin (2148), E18–19 (2343), E20–21 (2317), lactators (2165) and SON: virgin (2406), E18–19 (2466), E20–21 (2274), lactators (2466) (n = 6 animals).

### SON lysate preparation

The rats (n = 8 per group) were deeply anaesthetised with sodium pentobarbital (50 mg/kg) and perfused through the heart with cold sucrose solution (in mm: 20 d‐glucose, 0.45 ascorbic acid, 2.5 KCl, 1 MgSO_4_, 1.25 NaH_2_PO_4_.H_2_O, 26 NaHCO_3_, 210.35 sucrose, and 2 CaCl_2_). The brains were excised and sectioned coronally at 250 μm on a vibrating microtome (VT1000; Leica, Bannockburn, IL, USA). Small pieces from three or four hypothalamic brain slices were cut containing the SON and the immediate surrounding area, then transferred to sterile vials containing lysis buffer (pH 7.4) (in mm: 20 Hepes, 150 NaCl, 0.01% octylphenoxypolyethoxyethanol) with 0.01% protease inhibitor (#P8340; Sigma‐Aldrich,) and 0.01% phosphatase inhibitor 2 (#P5726; Sigma‐Aldrich,) cocktails. These SON pieces would include small portions of adjacent regions, in particular the perinuclear zone dorsally, the optic tract and chiasm medially, and the ventral anterior amygdaloid area and medial amygdaloid nucleus laterally 21. These pieces were homogenised manually by mortar and pestle and were passed three times through a 19‐gauge needle, and then a 25‐gauge needle followed by vortex. The homogenised lysates were then centrifuged at 14 489 g for 15 min and the supernatant was transferred to a fresh sterile vial, followed by protein estimation with BCA protein assay kit (Pierce, Thermo Scientific, Waltham, MA, USA). The entire process of lysate preparation was carried out on ice.

### Western blotting

Protein obtained from each SON‐lysate (100 μg) was mixed with 5–10 μl of 2 × Laemmli sample buffer and boiled at 95 °C for 5 min and the proteins were separated on 10–12% sodium dodecyl suphate (SDS)‐polyacrylamide gel electrophoresis (PAGE). Proteins were transferred to nitrocellulose membranes in electrophoresis buffer (25 mm Tris, 200 mm glycine and 20% methanol) for 120 min at 95 V at +4 °C. The membranes were then blocked either with 5% bovine serum albumin or 5% nonfat dry milk in Tris‐buffered saline containing (in mm: 1000 Tris, 50 NaCl, pH 8.0 adjusted with HCl and 0.001% Tween 20) (TBST) for 60 min at room‐temperature. The primary antibodies to either ERK1/2 or pERK1/2 (Cell Signaling) (dilution 1 : 1000) were added to the blocking buffer and were incubated overnight with the blot at +4 °C. The pERK1/2 antibody was the same rabbit anti‐pERK1/2 used for double‐labelling OT neurones for immunohistochemistry. The blots were washed three times with TBST buffer and incubated 60 min with goat anti‐mouse/rabbit (horse radish peroxidase‐conjugated) secondary antibody diluted in the blocking buffer at room temperature. They were washed three times with wash buffer and visualised with enhanced chemiluminescence reagents (Pierce‐ECL Western Blotting Substrate; Thermo Scientific) using Classic BX autoradiography film (MIDSCI, Valley Park, MO, USA). Immunoblot densities from film were scanned at 300 dpi, then analysed using imagej. A lysate from one animal from each group was run in one of the four lanes on a gel (one lane per group, virgin, E18–19, E20–21 and lactators). The experiment was repeated eight times (n = 8 for each group). After first processing and imaging for pERK1/2, these gels were stripped by incubating for 30 min at +50 °C in 30 ml of stripping buffer (2% SDS, 62.5 mm Tris HCL, pH 6.7 and 210 μl of β‐mercaptoethanol), then re‐probed for ERK1/2. For statistical comparisons, pERK1/2 values were normalised against ERK1/2 for each run. To control for variability in exposures across the eight runs, the pERK1/2/ERK1/2 ratios were ranked within each of the eight runs.

### Statistical analysis

The data were analysed with jmp pro, version 12 (SAS Institute Inc., Cary, NC, USA). For the pERK co‐localisation studies with VP or OT, we first tested for normality using the Shapiro–Wilk test and homogeneity of variance using Bartlett's test. In all but one case, we could not reject the null hypotheses that the distributions were normal or that the variances were equal (based on P < 0.05), and we therefore applied a standard anova followed by the Tukey–Kramer post‐hoc test. The exception to this was the co‐localisation data for VP with pERK in the PVN, which, although normal, did not satisfy the homogeneity of variance requirement (P = 0.019). These data were therefore analysed with Welch's test instead. For the western blot data, based on ordinal rankings, we used the nonparametric Kruskal–Wallis test followed by the Steel–Dwass test for between‐group comparisons.

## Results

### Increased pERK1/2 in OT neurones of the PVN and SON in late pregnancy

**Figure 1:**
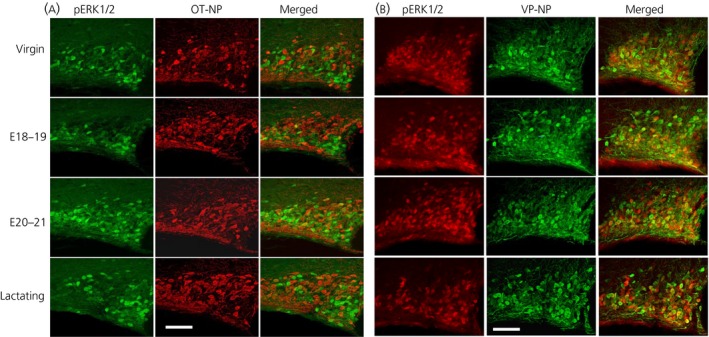
Immunolocalisation of phosphorylated ERK1/2 (pERK1/2) in oxytocin (OT) (a) and vasopressin (VP) (b) neurones in the supraoptic nucleus (SON) in virgin, embryonic day (E)18–19, E20‐21 and lactating rats. Double‐immunofluorescence confocal microscopy revealed that OT neurones (a), expressed more pERK1/2 during late pregnancy (E20‐21) than at other times. VP neurones co‐localised pERK1/2 to the same degree in all groups. Yellow cells/neurones in the merged columns represent double‐labelled neurones. Quantitative data are shown in Fig. 2. Scale bar = 100 μm.

**Figure 2:**
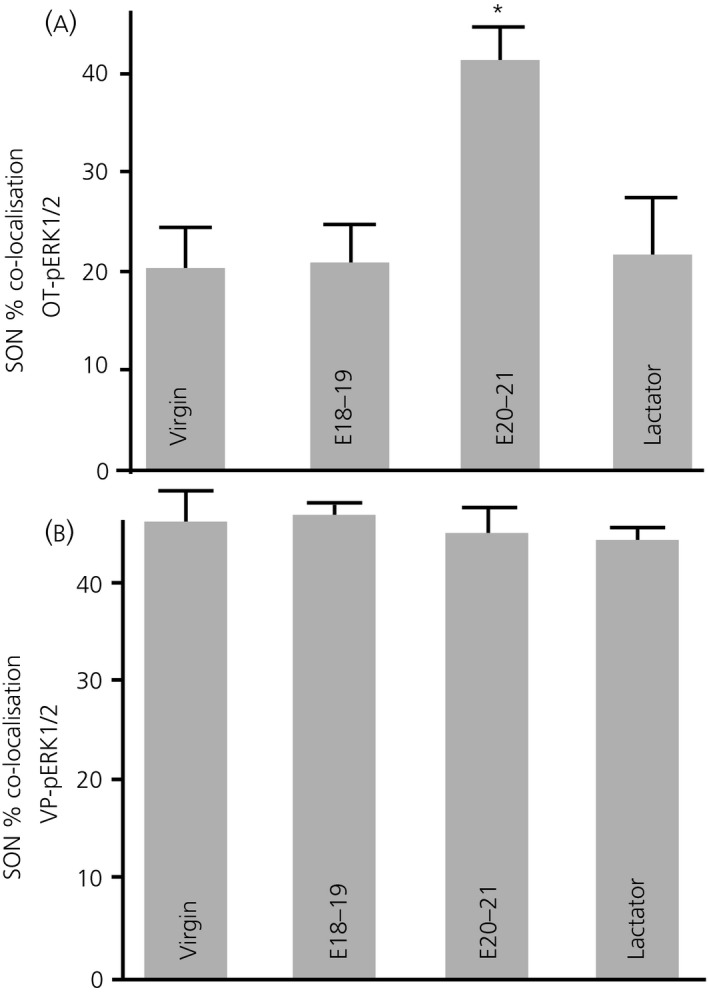
Co‐localisation of phosphorylated ERK1/2 (pERK1/2) with oxytocin (OT) (a) and vasopressin (VP) (b) in the supraoptic nucleus (SON) of virgin, embryonic day (E)18–19, E20–21 and lactating rats. anova indicated significant difference across groups for pERK1/2 co‐localisation with OT (P = 0.006) but not VP neurones (P = 0.835). Co‐localisation was highest at E20‐21 (*), which was significantly different from virgin (P = 0.017), E18–19 (P = 0.014) and lactating rats (P = 0.019) (n = 6 animals per group).

**Figure 3:**
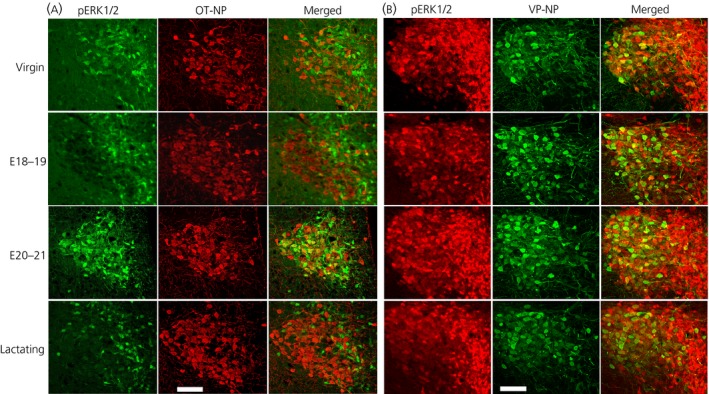
Immunolocalisation of phosphorylated ERK1/2 (pERK1/2) in oxytocin (OT) (a) and vasopressin (VP) (b) neurones in the paraventricular nucleus (PVN) in virgin, embryonic day (E)18–19, E20–21 and lactating rats. Double‐immunofluorescence confocal microscopy revealed that OT (a) but not VP (b) increased its co‐localisation with pERK1/2 in late pregnancy (E20–21). Yellow cells/neurones in the merged columns represent double‐labelled neurones. Quantitative data are shown in Fig. 4. Also note that the level of the PVN is more rostral for OT neurones than VP neurones, and that, medial to VP neurones, large numbers of parvocellular pERK1/2 neurones were visible. Scale bar = 100 μm.

We aimed to investigate whether the known plasticity in OT neurones at the end of pregnancy was specifically associated with changes in pERK1/2 in the SON and PVN. Rat hypothalamic sections were labelled with pERK1/2 and OT‐NP antibodies. The expression of pERK1/2 in the SON (Fig. 1 a) and PVN (Fig. 3a) varied across the groups studied. Although OT neurones were sampled throughout both nuclei, most of the OT neurones in PVN were found in the medial magnocellular group (Fig. 3a). The percentage of OT neurones double‐labelled for pERK1/2 was calculated and is shown in Figs 2(a) (SON) and 4(a) (PVN). The results from both PVN and SON revealed a two‐fold increase in the co‐localisation (of OT and pERK1/2) among late‐pregnant rats (E20‐21) compared to all the other three groups studied.

### Immunohistochemistry of ERK1/2 and VP neurones

**Figure 4:**
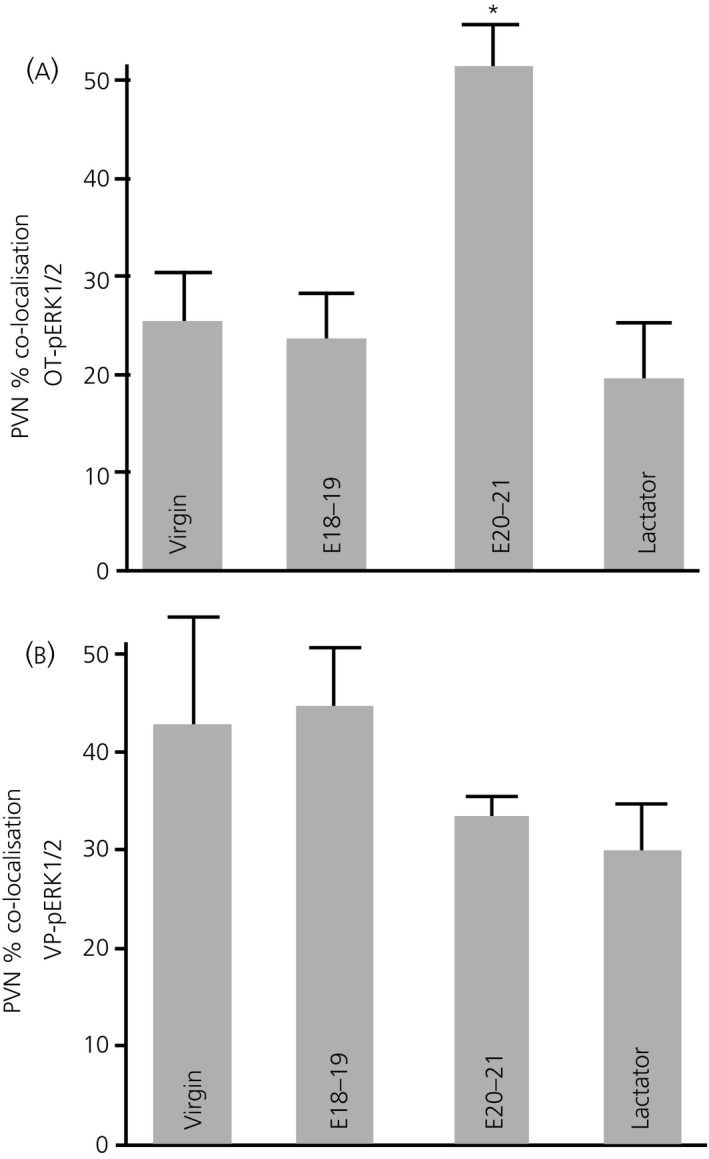
Co‐localisation of phosphorylated ERK1/2 (pERK1/2) in oxytocin (OT) (a) and vasopressin (VP) (b) neurones in the paraventricular nucleus (PVN) in virgin, embryonic day (E)18–19, E20–21 and lactating rats. anova indicated significant difference across groups for pERK1/2 co‐localisation with OT (P = 0.001) but not VP neurones (Welch's test, P = 0.216). Co‐localisation of OT‐pERK1/2 was highest at E20–21(*), which was significantly different from virgin (P = 0.008), E18–19 (P = 0.006) and lactating (P = 0.002) rats. n = 6 animals per group.

In many previous studies, changes in the electrophysiological properties of SON neurones during the reproductive cycle were found to be specific to OT neurones 22. To determine whether changes in pERK1/2 were similarly specific to OT and not VP neurones, sections of rat hypothalamus were reacted with a mouse pERK1/2 antibody and rabbit antibodies raised against VP‐NP. The mouse pERK1/2 antibody stained the SON and PVN in similar fashion to the rabbit antibody used, and gave identical bands in western blots (not shown). When sampling throughout both nuclei, in the PVN, most of the VP neurones were found in the lateral wing, just caudal to where most of the OT neurones were found (Fig. 3 b). Examples of such double‐staining are shown in Figs 1(b) and 3(b). No significant differences were found in co‐localisation in either the SON (Fig. 2 b) or the PVN (Fig. 4 b). However, the two nuclei appeared to behave differently in late pregnancy and lactation, where co‐localisation in the PVN trended downward (Fig. 4 b). Regardless, no significant changes were found in VP neurones. In the PVN, we also noted large numbers of pERK1/2 neurones in the medial, parvocellular portion of the PVN, immediately adjacent to the lateral wing of the VP‐rich portion of the PVN (Fig. 3 b). At the level of most of the OT neurones (which is rostral and medial to the largest cluster of VP neurones), these parvocellular pERK1/2 expressing neurones were less obvious (Fig. 3 a).

### Enhanced pERK1/2 activation in the late pregnant and lactating rats

**Figure 5:**
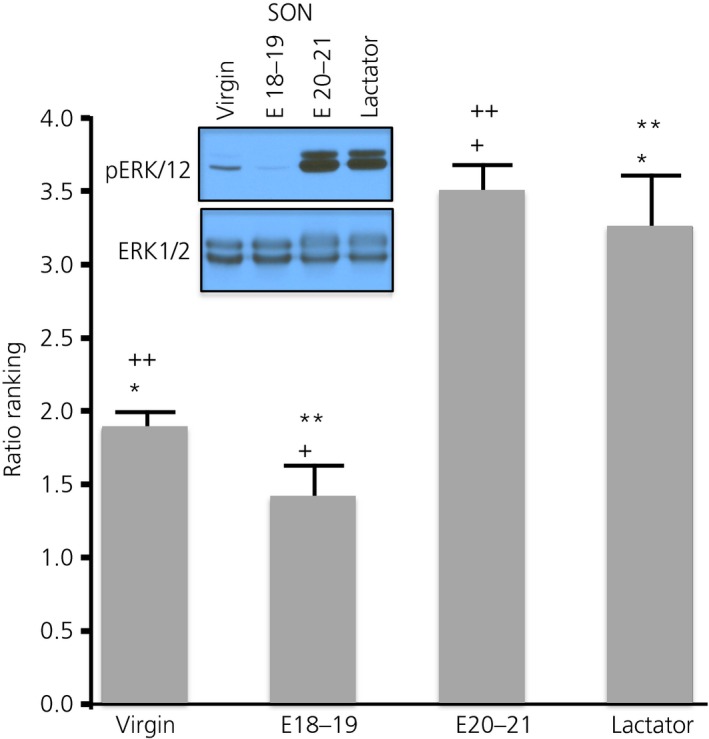
Phosphorylated ERK1/2 (pERK1/2) activation in supraoptic nucleus (SON) of embryonic day (E)20–21 and lactating rats. Histograms of the ranking of the ratio of pERK1/2 over ERK1/2 intensity for each of eight runs (n = 8 animals), with each run including lysates from one of the four groups. Inset: 100 μg of protein sample from hypothalamic SON lysates were probed on western blots (inset) with pERK1/2‐antibody (upper panel, inset) and the lower panel shows the blots used for normalisation with ERK1/2 antibody. A Kruskal–Wallis test indicated a difference among the groups (P = 0.0002). Comparisons between all pairs with Steel–Dwass indicated: *Lactator versus Virgin (P = 0.0387). **Lactator versus E18–19 (P = 0.0274). ^+^E20–21 versus E18–19 (P = 0.0062). ^++^E20–21 versus Virgin (P = 0.0025).

Because it is a more homogenous population of magnocellular neurones compared to the complex PVN (i.e. note the large numbers of parvocellular pERK1/2 neurones in the PVN in Fig. 3 b), protein lysates were analysed from the SON of virgin, pregnant (E18–19), late pregnant (E20–21) and lactating (day 8) rats. A significant increase in the activation of pERK1/2 was observed in the SON‐protein lysates of late pregnant (E20–21) rats compared to those at E18–19 (Fig. 5). However, in contrast to the immunohistochemical results, pERK1/2 levels remained significantly higher in lactators compared to E18–19 rats. These results suggested that pERK1/2 in regions immediately adjacent to the SON may be differentially regulated compared to OT neurones. To further investigate this issue, we examined histological sections for additional pERK1/2 neurones near the SON that might contribute to this difference. The largest groups of pERK1/2 neurones near the SON that might have been partially sampled with western blots were dorsally in the perinuclear zone, immediately adjacent to the anterior amygdala rostrally and the medial nucleus of the amygdala and the bed nucleus of the olfactory tract more caudally. We examined these regions in all animals, and could see no obvious, consistent difference in the number of cells and/or the intensity of labelling that might account for this particular difference.

## Discussion

In the present study, we found an up‐regulation of pERK1/2 expression in OT neurones during late pregnancy, 24–48 h prior to expected delivery, a time associated with hormonal changes that are considered critical to the induction of maternal behaviour and OT neuronal plasticity. Bridges 15 first showed that, although both progesterone and oestrogen gradually increase during pregnancy, approximately 2 days before parturition, progesterone levels dramatically decrease. In steroid replacement experiments in ovariectomised rats, the withdrawal of progesterone in the presence of oestrogen produces an increase in maternal behaviour 15, an up‐regulation of OTR binding 8 and increased OT mRNA 23. Similarly, in intact animals, this late gestational period and lactation are also associated with an up‐regulation of OT mRNA, OTR mRNA, OTR binding, and significant morphological and synaptic plasticity in magnocellular neurones 1. These events are also accompanied by increased somatodendritic OT release 9, 24. Thus, the fall of progesterone likely triggers dramatic changes in OT neurones, and our data show that the induction of pERK1/2 specifically within OT neurones in the PVN and SON is temporally associated with the onset of changes in OT neurone morphology and electrophysiology.

Changes in pERK1/2 within OT neurones are of particular interest because the local release of OT itself is implicated in the development of reproductive plasticity, probably by acting directly on OT neurones 12, 14, 22, 25, 26. In addition to the canonical role that MAPK pathways play in cell growth and differentiation 27, induction of pERK1/2 occurs in mature neurones and is associated with synaptic plasticity, both acutely as an intracellular messenger and by targeting nuclear transcription factors 28. Although OTR activation is classically G‐protein coupled and most often associated with increased intracellular calcium, it also leads to pERK1/2 signalling 16, 29, 30. Central OTR activation and its induction of pERK1/2 have been associated with range of functions, including spatial memory in the hippocampus of lactating rats 17 and anxiolysis mediated through the PVN 18, 30, 31. It was recently demonstrated that pERK1/2 is induced in both OT and VP neurones in response to hyperosmotic challenge 19. Indeed, pERK1/2 induction appears necessary for the acute membrane potential changes that MNCs demonstrate to direct osmotic challenge *in vitro,* and pERK1/2 levels in the SON are decreased below normal in response to hypotonic challenge, suggesting a constitutive role for pERK1/2 19. Furthermore, both psychological and systemic stressors also activate cytoplasmic pERK1/2 in an interleukin‐6‐containing subset of magnocellular VP neurones, as well as in parvocellular PVN neurones 20, suggesting that MAPK signalling pathways serve a variety of roles in the SON and PVN.

We also examined pERK1/2 by western blotting and, although again finding a significant increase in the SON at late pregnancy, levels did not return with lactation. However, these two methods measure different endpoints. It is possible that pERK1/2 may change its amount within individual neurones, without contributing to a change in double‐labelling. This caveat would include VP neurones, where co‐localisation with pERK1/2 did not change in these groups. In addition, there are regions adjacent to the SON (e.g. the perinuclear zone and the most medial aspects of the amygdala) that have some pERK1/2 neurones and which might contribute to the elevation during lactation, although these populations were unlikely to be consistently sampled to a large degree.

Morphological plasticity is induced by central OT administration 14, 25, as is the specialised bursting pattern that OT neurones exhibit during parturition and lactation 6, 32. The specialised bursting observed during lactation is also suppressed by OTR antagonism 6. Although probably not exclusively, the relevant central OTRs likely include those found on OT neurones themselves in the SON and PVN 33, 34 and these are likely activated by somatodendritically released OT 35, 36, 37. Further evidence of the role of OT in this regard is its ability to induce synaptic plasticity 38 and bursting *in vitro* 39. In our own laboratory, we found that plasticity in the calcium‐dependent afterhyperpolarisations of OT neurones is dependent on central OTR activation during pregnancy 12 and also that OT can induce this change *in vitro* in slices from pregnant rats, specifically in OT neurones 22. Although the present study does not directly address the role of pERK1/2 in OT plasticity, its elevation specifically within OT neurones during this critical period of late pregnancy suggests that pERK1/2 induction could be important for the dynamic changes that OT neurones will soon undergo in preparation for enhanced secretion during parturition, and lactation.

## References

[1] Armstrong WE . Central nervous system control of oxytocin secretion during lactation In: Plant TM, Zelezkni AJ, eds. Knobil and Neill's Physiology of Reproduction, 4th edn, Amsterdam: Elsevier/Academic Press, 2015, 527–563.

[2] Raggenbass M , Alberi S , Zaninetti M , Pierson P , Dreifuss JJ . Vasopressin and oxytocin action in the brain: cellular neurophysiological studies. Prog Brain Res 1998; 119: 263–273. 10074793 10.1016/s0079-6123(08)61574-5

[3] Insel TR . The challenge of translation in social neuroscience: a review of oxytocin, vasopressin, and affiliative behavior. Neuron 2010; 65: 768–779. 20346754 10.1016/j.neuron.2010.03.005 PMC2847497

[4] Pow DV , Morris JF . Dendrites of hypothalamic magnocellular neurons release neurohypophysial peptides by exocytosis. Neuroscience 1989; 32: 435–439. 2586758 10.1016/0306-4522(89)90091-2

[5] Ludwig M , Sabatier N , Bull PM , Landgraf R , Dayanithi G , Leng G . Intracellular calcium stores regulate activity‐dependent neuropeptide release from dendrites. Nature 2002; 418: 85–89. 12097911 10.1038/nature00822

[6] Lambert RC , Moos FC , Richard P . Action of endogenous oxytocin within the paraventricular or supraoptic nuclei: a powerful link in the regulation of the bursting pattern of oxytocin neurons during the milk‐ejection reflex in rats. Neuroscience 1993; 57: 1027–1038. 8309542 10.1016/0306-4522(93)90046-i

[7] Rossoni E , Feng J , Tirozzi B , Brown D , Leng G , Moos F . Emergent synchronous bursting of oxytocin neuronal network. PLoS Comput Biol 2008; 4: e1000123. 18636098 10.1371/journal.pcbi.1000123 PMC2440533

[8] Bealer SL , Lipschitz DL , Ramoz G , Crowley WR . Oxytocin receptor binding in the hypothalamus during gestation in rats. Am J Physiol Regul Integr Comp Physiol 2006; 291: R53–R58. 16832906 10.1152/ajpregu.00766.2005

[9] Neumann I , Russell JA , Landgraf R . Oxytocin and vasopressin release within the supraoptic and paraventricular nuclei of pregnant, parturient and lactating rats: a microdialysis study. Neuroscience 1993; 53: 65–75. 8469313 10.1016/0306-4522(93)90285-n

[10] Lipschitz DL , Crowley WR , Bealer SL . Central blockade of oxytocin receptors during late gestation disrupts systemic release of oxytocin during suckling in rats. J Neuroendocrinol 2003; 15: 743–748. 12834434 10.1046/j.1365-2826.2003.01052.x

[11] Bealer SL , Armstrong WE , Crowley WR . Oxytocin release in magnocellular nuclei: neurochemical mediators and functional significance during gestation. Am J Physiol Regul Integr Comp Physiol 2010; 299: R452–R458. 20554931 10.1152/ajpregu.00217.2010 PMC3774470

[12] Teruyama R , Lipschitz DL , Wang L , Ramoz GR , Crowley WR , Bealer SL , Armstrong WE . Central blockade of oxytocin receptors during mid‐late gestation reduces amplitude of slow afterhyperpolarization in supraoptic oxytocin neurons. Am J Physiol Endocrinol Metab 2008; 295: E1167–E1171. 18812459 10.1152/ajpendo.90620.2008 PMC2584811

[13] Theodosis DT . Oxytocin‐secreting neurons: a physiological model of morphological neuronal and glial plasticity in the adult hypothalamus. Front Neuroendocrinol 2002; 23: 101–135. 11906204 10.1006/frne.2001.0226

[14] Montagnese C , Poulain DA , Theodosis DT . Influence of ovarian steroids on the ultrastructural plasticity of the adult rat supraoptic nucleus induced by central administration of oxytocin. J Neuroendocrinol 1990; 2: 225–231. 19210388 10.1111/j.1365-2826.1990.tb00855.x

[15] Bridges RS . A quantitative analysis of the roles of dosage, sequence, and duration of estradiol and progesterone exposure in the regulation of maternal behavior in the rat. Endocrinology 1984; 114: 930–940. 6697968 10.1210/endo-114-3-930

[16] Zingg HH , Laporte SA . The oxytocin receptor. Trends Endocrinol Metab 2003; 14: 222–227. 12826328 10.1016/s1043-2760(03)00080-8

[17] Tomizawa K , Iga N , Lu YF , Moriwaki A , Matsushita M , Li ST , Miyamoto O , Itano T , Matsui H . Oxytocin improves long‐lasting spatial memory during motherhood through MAP kinase cascade. Nat Neurosci 2003; 6: 384–390. 12598900 10.1038/nn1023

[18] Jurek B , Slattery DA , Maloumby R , Hillerer K , Koszinowski S , Neumann ID , van den Burg EH . Differential contribution of hypothalamic MAPK activity to anxiety‐like behaviour in virgin and lactating rats. PLoS ONE 2012; 7: e37060. 22615888 10.1371/journal.pone.0037060 PMC3355176

[19] Dine J , Ducourneau VR , Fenelon VS , Fossat P , Amadio A , Eder M , Israel JM , Oliet SH , Voisin DL . Extracellular signal‐regulated kinase phosphorylation in forebrain neurones contributes to osmoregulatory mechanisms. J Physiol 2014; 592: 1637–1654. 24492838 10.1113/jphysiol.2013.261008 PMC3979616

[20] Jankord R , Zhang R , Flak JN , Solomon MB , Albertz J , Herman JP . Stress activation of IL‐6 neurons in the hypothalamus. Am J Physiol Regul Integr Comp Physiol 2010; 299: R343–R351. 20427720 10.1152/ajpregu.00131.2010 PMC2904148

[21] Paxinos G , Watson C . Paxinos and Watson's The Rat Brain in Stereotaxic Coordinates. 6th edn San Diego, CA: Academic Press Elsevier, 2006.

[22] Armstrong WE , Wang L , Li C , Teruyama R . Performance, properties and plasticity of identified oxytocin and vasopressin neurones in vitro. J Neuroendocrinol 2010; 22: 330–542. 20210845 10.1111/j.1365-2826.2010.01989.x PMC2910405

[23] Crowley RS , Insel TR , O'Keefe JA , Kim NB , Amico JA . Increased accumulation of oxytocin messenger ribonucleic acid in the hypothalamus of the female rat: induction by long term estradiol and progesterone administration and subsequent progesterone withdrawal. Endocrinology 1995; 136: 224–231. 7828535 10.1210/endo.136.1.7828535

[24] Moos F , Poulain DA , Rodriguez F , Guerné Y , Vincent JD , Richard P . Release of oxytocin within the supraoptic nucleus during the milk ejection reflex in rats. Exp Brain Res 1989; 76: 593–602. 2792248 10.1007/BF00248916

[25] Theodosis DT , Montagnese C , Rodriguez F , Vincent JD , Poulain DA . Oxytocin induces morphological plasticity in the adult hypothalamo‐neurohypophysial system. Nature 1986; 322: 738–740. 3748154 10.1038/322738a0

[26] Antonijevic IA , Russell JA , Bicknell RJ , Leng G , Douglas AJ . Effect of progesterone on the activation of neurones of the supraoptic nucleus during parturition. J Reprod Fertil 2000; 120: 367–376. 11058452 10.1530/jrf.0.1200367

[27] Pearson G , Robinson F , Beers Gibson T , Xu BE , Karandikar M , Berman K , Cobb MH . Mitogen‐activated protein (MAP) kinase pathways: regulation and physiological functions. Endocr Rev 2001; 22: 153–183. 11294822 10.1210/edrv.22.2.0428

[28] Thomas GM , Huganir RL . MAPK cascade signalling and synaptic plasticity. Nat Rev Neurosci 2004; 5: 173–183. 14976517 10.1038/nrn1346

[29] Gimpl G , Fahrenholz F . The oxytocin receptor system: structure, function, and regulation. Physiol Rev 2001; 81: 629–683. 11274341 10.1152/physrev.2001.81.2.629

[30] Blume A , Bosch OJ , Miklos S , Torner L , Wales L , Waldherr M , Neumann ID . Oxytocin reduces anxiety via ERK1/2 activation: local effect within the rat hypothalamic paraventricular nucleus. Eur J Neurosci 2008; 27: 1947–1956. 18412615 10.1111/j.1460-9568.2008.06184.x

[31] Neumann ID . Brain oxytocin: a key regulator of emotional and social behaviours in both females and males. J Neuroendocrinol 2008; 20: 858–865. 18601710 10.1111/j.1365-2826.2008.01726.x

[32] Moos F , Richard P . Paraventricular and supraoptic bursting oxytocin cells in rat are locally regulated by oxytocin and functionally related. J Physiol 1989; 408: 1–18. 2778722 10.1113/jphysiol.1989.sp017442 PMC1190386

[33] Freund Mercier MJ , Stoeckel ME , Klein MJ . Oxytocin receptors on oxytocin neurones: histoautoradiographic detection in the lactating rat. J Physiol 1994; 480: 155–161. 7853219 10.1113/jphysiol.1994.sp020349 PMC1155786

[34] Lambert RC , Dayanithi G , Moos FC , Richard P . A rise in the intracellular Ca2+ concentration of isolated rat supraoptic cells in response to oxytocin. J Physiol 1994; 478: 275–287. 7525943 10.1113/jphysiol.1994.sp020249 PMC1155685

[35] Moos F , Freund Mercier MJ , Guerne Y , Guerne JM , Stoeckel ME , Richard P . Release of oxytocin and vasopressin by magnocellular nuclei in vitro: specific facilitatory effect of oxytocin on its own release. J Endocrinol 1984; 102: 63–72. 6539805 10.1677/joe.0.1020063

[36] Ludwig M . Dendritic release of vasopressin and oxytocin. J Neuroendocrinol 1998; 10: 881–895. 9870745 10.1046/j.1365-2826.1998.00279.x

[37] Ludwig M , Leng G . Dendritic peptide release and peptide‐dependent behaviours. Nat Rev Neurosci 2006; 7: 126–136. 16429122 10.1038/nrn1845

[38] Langle SL , Poulain DA , Theodosis DT . Induction of rapid, activity‐dependent neuronal‐glial remodelling in the adult rat hypothalamus in vitro. Eur J Neurosci 2003; 18: 206–214. 12859353 10.1046/j.1460-9568.2003.02741.x

[39] Israel J‐M , Poulain DA . Oxytocin neurons during suckling: lessons from organotypic cultures In: Armstrong WE, Tasker JG, eds. Neurophysiology of Neuroendocrine Neurons. Oxford: John Wiley & Sons, 2014: 29–62.

